# Oxidative Mechanism of Peyronie’s Disease and Effectiveness of Pentoxifylline in the Therapeutic Management: A Narrative Review

**DOI:** 10.3390/antiox14020208

**Published:** 2025-02-12

**Authors:** Gianni Paulis, Andrea Paulis

**Affiliations:** 1 Department of Urology and Andrology, Peyronie’s Care Center, Castelfidardo Clinical Analysis Center, 00185 Rome, Italy; 2 Bambino Gesu’ Children’s Hospital, IRCCS [Istituto di Ricovero e Cura a Carattere Scientifico], 00165 Rome, Italy; andrea.fx.94@gmail.com

**Keywords:** Peyronie’s disease, oxidative stress, pentoxifylline, antioxidants

## Abstract

Peyronie’s disease (PD) is a chronic disease characterized by the development of fibrous tissue in the tunica albuginea of the penile corpora cavernosa that causes penile deformity. The precise cause of PD is not completely understood, but it is generally believed to be initiated by a specific injury in the affected area. Research has consistently shown that oxidative stress (OS) is a key player in PD. Pentoxifylline (PTX) is a synthetic derivative of methylxanthine that was initially used for the management of peripheral vascular disease. PTX has also been used in humans for several inflammatory and fibrotic conditions, including PD. PTX has several mechanisms of action, including antioxidant, antifibrotic, anti-inflammatory, and vasorelaxant. This article aims to verify, after a review of the literature regarding the use of PTX in PD, whether this substance is really able to cure PD. We conducted research by consulting the scientific literature on the topic. Results: After examining 39 articles, we considered 20 articles eligible for our narrative review, including a single randomized controlled clinical study, six clinical studies with a control group, a single uncontrolled clinical study, eight case report studies, and four systematic review articles. Conclusions: Although the systematic review articles selected in this paper showed no consistent evidence regarding the efficacy of PTX, in our opinion, the clinical studies we have analyzed undoubtedly demonstrate that PTX is able to combat PD, thanks to its ability to interfere with the pathogenic mechanisms of the disease. However, we believe that further new randomized controlled trials are necessary to more clearly demonstrate the effectiveness of PTX in the treatment of PD.

## 1. Introduction

Peyronie’s disease (PD) is a benign condition marked by the formation of fibrous tissue in the tunica albuginea of the penile corpora cavernosa. PD is a genetically based disease that affects some males, resulting in the development of a plaque in the penis that ultimately leads to various forms of penile deformity [1,2]. PD is transmitted genetically in an autosomal-dominant manner, and familial aggregation and genetic transmission through HLA-B7 antigens have been previously described [2,3]. The WNT2 genetic locus has also been identified, which is involved in the genetic predisposition for both PD and Dupuytren’s disease [4]. The prevalence of PD varies from 3.2% to 13.1% and is less frequently seen in Asian countries, with rates of about 0.6% in Japan [5,6,7,8,9]. These differences are probably related to environmental, genetic, and lifestyle factors. It is thought that the prevalence of PD in males is even higher due to the underestimation of symptoms by patients, as they may feel uncomfortable and decide not to report their problem to the doctor [10]. Symptoms of PD include penile deformity, penile pain, erectile dysfunction (ED), and an anxious–depressive state. These symptoms have a devastating effect on a person’s physical and psychological well-being. Penile deformity may present as curvature, shortening, torsion, indentation, or an hourglass penis [11,12,13,14,15]. The exact cause of PD is not fully understood, but it is commonly thought to be triggered by a specific injury in the affected area [16,17,18]. Although PD can be triggered by coital sexual trauma or injury, in 70% of cases, no specific cause is found, and patients do not remember the traumatic event [19]. Following an injury, fibrin is deposited, and a hematoma forms. In males without a genetic predisposition to the disease, the hematoma in the penile corpora cavernosa is completely reabsorbed within a few days. However, in individuals with a genetic predisposition, the hematoma that forms triggers the activation of inflammatory cells and pro-inflammatory cytokines, leading to the subsequent formation of an area of chronic inflammatory tissue that inevitably progresses to fibrosis (plaque) [20,21,22]. This plaque becomes progressively less and less elastic. Histologically, the Peyronie’s plaques are characterized by a disorganization of collagen associated with a reduction in the density of elastin [23]. PD is divided into two stages: (1) the acute inflammatory phase (active and remodeling phase) and (2) the stabilization phase. The first phase of PD is characterized by a short acute post-traumatic period lasting about two weeks. During this period, after blood extravasation and fibrin accumulation, there is a strong recruitment of macrophages, inflammatory cells, and lymphocytes [24]. During this short period, no penile nodules can be felt, and no plaque can be observed by ultrasound [21]. This short period is followed by an inflammatory remodeling phase, lasting approximately 12–18 months [24,25]. At the conclusion of this first stage, the disease tends to stabilize. In the second phase (PD stabilization), the penile pain has typically disappeared, the penile deformation is stable, and the fibrosis and/or the calcification of the plaque is already completed, making the penile nodule definitely palpable [21,22]. The plaque evolves into calcification only in 20–25% of cases [26].

Conservative medical treatment is recommended for the first phase of PD, while surgery is recommended for the second phase of the disease [24,27,28]. Conservative medical treatment includes oral therapies, penile infiltrations, and physical treatments. Oral therapies include vitamin E; colchicine; tamoxifen; pentoxifylline (PTX); antioxidants (e.g., carnitine, L-arginine, bilberry, propolis, and coenzyme Q10); phosphodiesterase-5 inhibitors (PDE5is); and non-steroidal anti-inflammatory drugs (NSAIDs). Penile infiltrations are performed with verapamil, interferon-α2b (IFNα2b), corticosteroids, hyaluronic acid, PTX, and collagenase clostridium histolyticum (CCH) [24,27,29,30,31,32,33]. Physical treatments consist of iontophoresis, extracorporeal shock wave therapy (ESWT), and penile vacuum and penile traction devices [24,29,34,35].

## 2. Relevant Sections

This article illustrates the pathogenetic mechanisms of PD and provides an overview of the current scientific literature on the use of pentoxifylline (PTX) in the conservative treatment of PD.

### 2.1. Pathogenetic Mechanisms and Oxidative Stress in PD

Numerous studies have demonstrated that oxidative stress (OS) is a crucial factor in all types of inflammatory processes, whether they are acute or chronic [36,37,38,39,40]. Research has consistently shown that OS is a key player in PD, a condition characterized by chronic inflammation in the penile corpora cavernosa [20,21,41,42,43,44,45,46,47,48,49,50]. The most important biological mediators in the pathogenesis of PD are as follows: reactive oxygen species (ROS), reactive nitrogen species (RNS), nuclear factor kappa-light-chain-enhancer of activated B cells (NF-κB), transforming growth factor beta-1 (TGF-ß1), platelet-derived growth factor (PDGF), interleukin-1 (IL-1), basic fibroblast growth factor (bFGF), plasminogen activator inhibitor-1 (PAI-1), and tumor necrosis factor-alpha (TNF-α).

ROS are generated in significant amounts by macrophages and neutrophil granulocytes, which are present in high concentrations in the disease area. The production of ROS triggers the activation of NF-κB, which controls DNA transcription and specifically regulates the expression of genes such as TGF-ß1, bFGF, fibrin, collagen, and inducible nitric oxide synthase (i-NOS), among others [21,22,42,51].

RNS are present in high levels due to the action of the i-NOS enzyme, produced locally due to inflammation. RNS play a role in promoting a higher oxidation state (nitro-oxidation), which leads to lipid peroxidation, DNA fragmentation, protein nitration, and vasoconstriction [21,22,49,52,53,54].

TGF-ß1 is a cytokine secreted by macrophages, platelets, T lymphocytes, and neutrophils. TGF-ß1 carries out the following actions: It attracts neutrophils, monocytes, lymphocytes, and fibroblasts; promotes fibroblast growth and their transformation into myofibroblasts; induces collagen production by fibroblasts; stimulates collagen synthesis and deposition; promotes osteogenesis in the PD plaque; and enhances the production of tissue inhibitors of matrix metalloproteinases (TIMP-1) [21,22].

PDGF is a cytokine generated by platelets, macrophages, endothelial cells, and fibroblasts. PDGF production is promoted by the local accumulation of fibrin. This cytokine carries out the following activities: It attracts fibroblasts to the site and promotes their proliferation and transformation into myofibroblasts, promotes collagen production and deposition, acts as a recruiter for osteoblasts and promotes calcification and ossification of the PD plaque, promotes the production of TIMP-1, and promotes the production of MMP-2, which has elastase activity [21,22].

### 2.2. Rationale for the Use of PTX in the Treatment of Peyronie’s Disease

PTX is a synthetic methylxanthine derivative that shares structural similarities with caffeine and theophylline. Initially developed as a hemorheological agent, it was first utilized in the management of peripheral vascular diseases, cerebrovascular insufficiency, diabetic neuropathy, and sickle cell disease [21]. PTX has also been used in humans (in divided doses of 800–1600 mg daily) for several inflammatory and fibrotic conditions, including cystic fibrosis, radiation proctitis, radiation pneumonitis, osteoradionecrosis, steatohepatitis, radiation fibrosis, and epidural fibrosis [55,56,57,58]. Brant et al. (2006) were the first to use PTX in PD, using the same dosage as in the previously mentioned conditions [59]. At that time, while the mechanism of action of PTX was not fully understood, it was known that PTX can block TGF-ß1-mediated inflammation, hinder type I collagen deposition, and act as a non-specific phosphodiesterase (PDE) inhibitor.

PTX has the following properties: antioxidant, antifibrotic, anti-inflammatory, anti-calcific, vasorelaxant, and antiplatelet actions [21,22,60,61,62,63,64,65,66,67,68,69]. More specifically, PTX acts through the mechanisms detailed in Table 1.

Although several guidelines do not recommend the use of antioxidants (including PTX) in the treatment of PD, many therapeutic experiences have been published with the use of PTX, either alone or in combination with other antioxidant and non-antioxidant therapies [27,70,71,72].

This article aims to verify, after a review of the literature regarding the use of PTX in PD, whether this substance is truly able to counteract the pathogenetic mechanisms of PD and reduce the symptoms of the disease (penile curvature, pain, and erectile dysfunction). The key questions identified for the review topic: “Peyronie’s disease and pentoxifylline treatment”.

## 3. Materials and Methods

To verify whether PTX is really able to cure PD, we conducted research by consulting the scientific literature on the topic “Peyronie’s disease and pentoxifylline treatment” using the following databases: Medline (PubMed), EMBASE, Google Scholar, and Cochrane Library. We searched for all articles published in English from 1 January 1980 to 29 September 2024.

Inclusion criteria: clinical studies, case reports, and case series where PTX has been used to treat PD in humans, as well as systematic review articles in which the therapeutic use of PTX and the results were explicitly discussed.

Exclusion criteria: narrative reviews and articles that do not focus solely on PD therapy with PTX, studies where PTX was utilized for the treatment of other penile conditions instead of PD, narrative review articles and studies where PTX has not been used to treat PD in humans (experimental studies), articles in which the therapeutic effect of PTX has not been analyzed, and studies with a retraction statement.

In all statistical studies related to clinical trials, a value of *p* < 0.05 was considered statistically significant.

## 4. Results

### Literature Review

After examining the 39 articles, we considered 20 articles eligible for our paper, including a single randomized controlled clinical study, six clinical studies with a control group, a single uncontrolled clinical study, eight case report studies, and four systematic review articles. We excluded 19 articles for the following reasons: in 10 cases, the articles were narrative reviews or simple articles in which not only PTX was mentioned as a therapeutic option; in 1 study, PTX was not used to treat PD but, instead, was used to treat other penile diseases; 2 experimental studies were on human material from patients suffering from PD; a single experimental study where PTX was administered to rats in which PD had been experimentally induced; 3 articles where the therapeutic effect of PTX was not studied; a double-blind, placebo-controlled study with a retraction statement; and a single systematic review was excluded because the assessment related to PTX referred to a study retracted from PubMed (retraction statement). 

**Figure 1:**
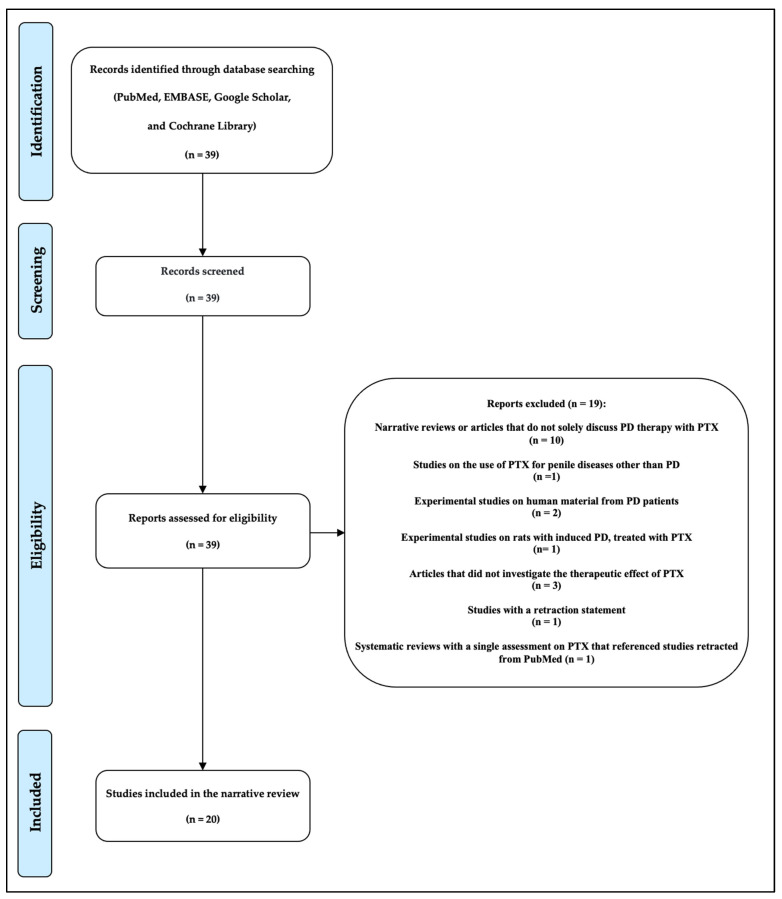
Explanatory flow chart of the narrative revision methodology.

Following a thorough review of the entire text, 20 articles were selected for the final review out of the initial 39 articles (Figure 1).

## 5. Discussion

Although several guidelines do not recommend the use of antioxidants (including PTX) in the treatment of PD, several clinical trials, case reports, and review articles have been published on the use of antioxidants, including PTX alone or in combination with other antioxidant substances or other treatments [27,35,59,70,71,72,73,74,75,76,77,78,79,80,81,82,83,84,85,86,87,88,89,90,91,92,93,94,95,96,97,98,99,100]. We must still point out that there are some conflicting views on this topic among the various international guidelines. For example, the guidelines of the Canadian Urological Association for Peyronie’s disease consider PTX as a possible therapeutic option, both as a monotherapy or in combination with other types of treatment, while acknowledging the limited evidence available on the treatment’s efficacy [101].

In most published clinical trials using antioxidant agents to treat PD, a multi-modal (or combination) therapy has been considered [22]. Therefore, antioxidant substances have been administered in combination with other substances or other types of treatments. The goal of multi-modal therapy is to achieve a better therapeutic result than that obtainable when administering a single substance. This approach can counteract PD and its various biochemical mechanisms by interfering in different ways with the numerous “chemical messengers” involved in the disease [21,22]. Multi-modal or combined treatment is a therapeutic approach that has been used in various medical fields, as well as for the treatment of PD [102], including oncology (polychemotherapy) and infection treatment (antibiotic combination therapy).

Of the 20 articles included in our narrative review, 7 were randomized or controlled clinical trials, 9 were uncontrolled studies or case reports, and 4 were systematic reviews that specifically discussed the use of PTX in addition to other treatment options.

The first article in which PTX was used to treat patients with PD was published by Brant et al. in 2006 [59]. It was a case report describing a patient with PD who, after undergoing oral therapy with PTX (400 mg orally, three times daily) for a period of 6 months, experienced improved penile curvature after 2 years and no longer suffered from erectile dysfunction. Additionally, one of the two calcifications of the plaque was shown to have regressed during the ultrasound examination. The authors concluded their article by stating that, despite the often disappointing results of conservative oral therapy for PD, in their case, the results after PTX therapy had been very satisfactory. This is a “historic” publication, as it inspired and encouraged many urologists and andrologists to use PTX to conservatively treat their patients with PD. In fact, by 2018, PTX was the most frequently prescribed oral medication for treating PD, with a usage rate of 33%. PTX is now the most commonly prescribed oral medication, having replaced colchicine, while injectable collagenase (CCH) is the most commonly administered substance by injection. PTX treatment is commonly used, as it is cost-effective, convenient, and has minimal side effects.

The study by Smith et al. also has historical significance, as it was the first controlled study to be published in which PTX was administered orally (as monotherapy) to treat patients with PD [73]. The authors noted some articles in the literature where PTX had improved certain chronic and fibrotic diseases [55,56,57,58,102]. They also referenced Brant’s study, which showed that PTX improved symptoms and ultrasound signs in a patient with PD. The results of Smith’s study were very important, as the administration of PTX (400 mg, orally three times daily) was able, after 1.2 years, to reduce the volume of plaque calcifications by an average of 69.4%. Furthermore, 91.9% of patients treated with PTX did not show an increase in calcium content upon ultrasound examination, and in some of them, the plaque calcium content was reduced.

This property of PTX is most likely due to its ability to reduce the synthesis of the pro-fibrotic cytokines TGF-ß1 and PDGF, which are capable of promoting osteogenesis in PD plaque, thus inducing its calcification [21,22,46,62,63,103,104].

Regarding the six clinical studies with a control group, including the only randomized controlled study, where PTX was used in combination with other treatments (Abern et al. 2012, Paulis et al. 2015, Paulis et al. 2017, Gallo et al. 2019, Topcuoglu et al. 2022, and Alizadeh et al. 2014), excellent results were achieved in reducing penile curvature in the single controlled and randomized study, as well as in all five controlled clinical trials [74,77,79,80,82,84]. In three of these studies, penile curvature was also analyzed and was always significantly reduced at the post-treatment controls. In five of these studies, erectile function significantly improved at the post-treatment controls, as well as a significant reduction in penile pain. In the remaining study, the endpoints mentioned above were not evaluated, only penile curvature, which, as mentioned above, significantly improved, along with penile length (SPL). The salient features of all the selected clinical trials are reported in Table 2 and Table 3.

The only uncontrolled study where PTX was used in combination with other treatments was that of Ibrahim et al. (2019), which involved 46 patients with PD [81]. The treatment consisted of a 6-month combination therapy, which included penile traction therapy (PTT), oral PTX, and oral colchicine (for patients experiencing penile pain). Statistical analysis revealed that PTT therapy, when combined with PTX or colchicine, can be effective in reducing penile curvature and plaque volume in patients with PD. The authors concluded the article by stating that oral therapy using PTX (or colchicine) combined with PTT can be considered as a potentially economical, convenient, and effective treatment for reducing penile curvature and plaque volume in subjects with PD. The features of this study are reported in Table 3.

In the six clinical studies with a control group, including the only randomized controlled study [74,77,79,80,82,84], PTX was used in combination with other treatments (multi-modal or combination therapy), and in all of these studies, this therapeutic approach was effective and able to achieve excellent statistically significant results. Even in these studies, it was shown that multi-modal therapy can achieve better results than the use of a single substance or treatment.

In the selected case reports in this review (Brant et al. 2006, Ciociola et al. 2013, Dell’Atti et al. 2014, and Paulis et al. 2022; 2024), excluding the study by Brant, PTX was administered in combination with other active substances or treatments [59,75,76,85,86,87,88,89]. Notably, in all these studies, excellent results were obtained.

Our research group has conducted five case report studies [85,86,87,88,89]. In 10 cases included in the five case report articles, PTX was administered through penile injections and not orally [85,86,87,88,89]. In all 15 patients, we used several antioxidants (see Table 4) and PTX for penile infiltration. In five cases, combined oral and topical therapy was not associated with infiltrative therapy with PTX, either because the plaque was not voluminous or because the patients did not consent to this type of administration.

In the other studies, substantial improvements were observed, such as a reduction in plaque volume in the experience of Ciociola et al., and the disappearance of calcification, reduction in curvature, and improvement in penile rigidity in the study conducted by Brant et al. [59,75]. The latter symptom also improved in the study conducted by Dell’Atti et al., where the patient had not reported any curvature of the penis either before or after treatment [76]. In all of these case report studies, the authors stated that, although the relevant scientific literature does not consider this conservative treatment sufficiently recommendable, the results were undoubtedly positive, as PTX proved to be able to counteract some pathogenetic mechanisms known and shared by the scientific literature regarding PD [68,69,70,104,105,106,107,108,109]. The main features of all the selected case reports where PTX was used to treat PD patients are reported in Table 4.

As we have already mentioned, in our most recent studies, we stopped administering PTX orally, as we detected a high percentage (15.7%) of side effects affecting the circulatory system; blood pressure; and the intestinal system (e.g., blood pressure drop, tachycardia, dizziness, headache, nausea, vomiting, and flatulence) [79]. Therefore, in our most recent studies, we used PTX only through perilesional penile injections, still obtaining undoubtedly good results [80,85,86,87,88,89]. We did not find any other studies in the literature where PTX penile injections had been used to treat PD. We only found one controlled study where PTX infiltrations were successfully used to treat alopecia [110].

All the systematic review articles selected in this paper (Verze et al. 2014, Chung et al. 2016, El-Sakka et al. 2021, and Hayat et al. 2023) showed no consistent evidence regarding the efficacy of PTX, and the authors did not consider this sufficiently recommendable for the treatment of PD [27,78,83,90].

We would like to point out that the only systematic review article we did not include in our review was the one related to the article by Lee et al. [111]. In fact, in this review that coincided with the exclusion criteria, the only reference to PTX as a therapeutic option for PD referred to an article withdrawn from PubMed by the same authors. In our opinion, the clinical studies we have analyzed demonstrate that PTX is able to combat PD, thanks to its ability to interfere with the pathogenic mechanisms of the disease. The studies have shown us that PTX is able to reduce the symptoms of the disease, including curvature, pain, and erectile dysfunction. In the studies where penile ultrasound examination was performed, after treatment, a reduction in plaque volume was demonstrated in all cases, and complete regression of the plaque was even observed in some of the case reports selected in our review.

We want to emphasize the novelty of our research on the conservative treatment of PD. In our studies, we have shown multiple times that it is not only possible to treat this disease but to regress it with the administration of oral antioxidants, combining them with the penile injection of pentoxifylline.

Most of the existing literature on the treatment of PD emphasizes the use of collagenase clostridium histolyticum (CCH) or corporoplasty intervention, with the aim of correcting penile curvature. We believe that this type of therapeutic approach represents only a “curvature therapy”, ultimately a therapy for a symptom and not for the disease (PD). Our therapeutic approach is instead based on treating the disease, and it is generally applied in all fields of medicine. We believe that our studies have sufficiently demonstrated that our conservative therapeutic approach is the most appropriate. In fact, we have shown cases of disease healing and the regression of PD symptoms.

The main limitation of our review is that it is not a systematic review following the PRISMA guidelines. Another limitation that significantly impacted our review is that, after searching for articles in the scientific literature, we only found one randomized controlled trial (RCT) and a few other controlled trials.

## 6. Conclusions and Future Directions

The management of PD remains not ideal, as there are many different treatments available, and a complete understanding of all the disease’s underlying mechanisms is still lacking. We believe that it is necessary to start from the current knowledge of the pathophysiology of PD and not focus on treating the symptom (curvature) but, instead, on the disease that caused it. The results of antioxidant therapy and, therefore, PTX demonstrate that therapeutically acting with drugs that interfere with the biochemical mechanisms of PD is the right path to follow. The therapeutic results observed after the use of PTX, as shown in this narrative review, seem to demonstrate its effectiveness. Unfortunately, in our review, we found only one RCT study. New RCTs on this topic are needed, in order to better demonstrate the effectiveness of PTX in PD treatment, and we ourselves will commit to this goal. At the same time, it is necessary to further investigate the pathophysiological aspects of PD to achieve more effective therapeutic results. For this reason, management by experienced urologists and andrologists is required in order to improve the outcomes for men with PD and their partners.

## Figures and Tables

**Table 1:** Biological properties and mechanisms of the actions of pentoxifylline.

Biochemical Property of Pentoxifylline (PTX)	Molecular Mechanism	References
**Antioxidant**	- Inhibits the respiratory/oxidative burst	[21,22,61,65,67]
	- Reduces the synthesis of i-NOS	
	- Eliminates ROS and RNS	
	- Inhibits lipid peroxidation	
**Anti-inflammatory**	- Reduces synthesis of TNF-α, IL-1, IL-6, IL-8, IL-10, PAI-1	[21,22,64,67]
	- Impedes inflammation by inhibiting the NF-κB factor	
	- Reduces synthesis of the cyclooxygenase-2 (COX-2) enzyme	
**Anti-fibrotic**	- Reduces the synthesis of TGF-ß1, PDGF, and PAI-1	[21,22,63,66]
	- Reduces collagen synthesis and deposition	
	- Stimulates fibroblast apoptosis	
	- Inhibits fibroblast proliferation and their transformation into myofibroblasts	
**Anti-calcifying**	- Reduces the synthesis of TGF-ß1 and PDGF, which promote osteogenesis and related calcification	[68,69]
**Vasorelaxant**	- Acts as a non-specific inhibitor of PDEs and determines vasodilation through a non-selective inhibition of PDEs, preventing the degradation of cAMP	[21,22,62]
**Anti-platelet**	- Inhibits platelet aggregation	[21,22,60]

Note: Note 1: i-NOS = inducible nitric oxide synthase; ROS = reactive oxygen species; RNS = reactive nitrogen species; TNF-*α* = tumor necrosis factor-α; IL-1 = interleukin-1; IL-6 = interleukin-6; IL-8 = interleukin-8; IL-10 = interleukin-10; NF-κB = nuclear factor kappa-light-chain-enhancer of activated B cells; TGF-ß1 = transforming growth factor-beta-1; PDGF = platelet-derived growth factor; PAI-1 = plasminogen activator inhibitor-1; PDEs = phosphodiesterase inhibitors; cAMP = cyclic adenosine monophosphate. Note 2: The various articles cited regarding the different properties of pentoxifylline refer to various types of studies: experimental studies on rats [63,64,67,68], studies on humans [60,62,65,66], an in vitro biochemical study [61], and review articles [21,22,64,69].

**Table 2:** Characteristics of the clinical trials included in the narrative review.

Authors (Year) and [Reference]	Smith et al. (2011) [73]	Abern et al. (2012) [74]	Alizadeh et al. (2014) [77]	Paulis et al. (2015) [79]
Study design	Retrospective, observational, controlled study	Prospective, controlled study	Prospective, randomized, and controlled study	Retrospective, controlled study
Treatment/control	PTX 400 mg orally, three times daily for a period of 1.2 years. *Versus* Placebo	PTX 1200 mg orally, daily + L-arginine orally 2 g daily + verapamil 10 mg penile infiltrations every 2 weeks (for a total of 12 injections) + daily penile traction therapy (PTT) for a period of 6 months. *Versus* The same oral and infiltrative therapy described above without PTT, for a period of 6 months.	PTX 400 mg orally, three times daily for a period of 6 months. *Versus* Verapamil 10 mg penile infiltrations every 2 weeks (for a total of 12 injections) for a period of 6 months. *Versus* Combination of the two therapies described above, for a period of 6 months.	PTX 400 mg orally, twice daily + other oral antioxidants + diclofenac 4% gel, twice daily + PTX 100 mg. penile infiltrations every 2 weeks (for a total of 12 injections) for a period of 6 months. *Versus* The same therapy as above, excluding infiltrations with PTX, for a period of 6 months. *Versus* Placebo for a period of 6 months.
Active or chronic phase	Chronic	ND	ND	Active phase
Follow-up period	1.2 years	6 months	6 months	6 months
Total number of patients	71	74	90	307
*N* treated	62	39	30	112 + 94
*N* control	9	35	30 + 30	101
Evidence for improvement? (YES/NO)				
Penile curvature	ND	YES	YES	YES
Penile pain	ND	ND	YES	YES
IIEF (Erectile function)	ND	ND	YES	YES
Penile length	ND	YES	ND	ND
Plaque size	ND	ND	YES	YES
Calcification	YES	ND	ND	YES

NOTE: PTX = pentoxifylline; PTT = penile traction therapy; ND = not defined.

**Table 3:** Characteristics of the clinical studies included in the narrative review.

Authors (Year) and [Reference]	Paulis et al. (2017) [80]	Ibrahim et al. (2019) [81]	Gallo et al. (2019) [82]	Topcuoglu et al. (2022) [84]
Study design	Retrospective, controlled study	Prospective, uncontrolled study	Retrospective, controlled study	Retrospective, controlled study
Treatment/control	Group A: Silymarin 200 mg orally, twice daily. Group B: Silymarin 200 mg orally, twice daily + Ginkgo biloba 250 mg orally, once daily. Group C: Silymarin 200 mg orally, twice daily + Ginkgo biloba 250 mg orally, once daily + Vitamin E 400 IU orally, twice daily. Group D: Silymarin 200 mg orally, twice daily + Ginkgo biloba 250 mg orally, once daily + Vitamin E 400 IU orally, twice daily + Propolis 600 mg orally, once daily + Vaccinium myrtillus 160 mg orally, once daily + Diclofenac sodium spray-gel 4% locally, two applications per day (2 pumps equivalent to 16 mg of diclofenac sodium). Group E: Silymarin 200 mg orally, twice daily + Ginkgo biloba 250 mg orally, once daily + Vitamin E 400 IU orally, twice daily + Propolis 600 mg orally, once daily + Vaccinium myrtillus 160 mg orally, once daily + Diclofenac sodium spray-gel 4% locally, two applications per day (2 pumps) + PTX 100 mg penile infiltrations every 2 weeks (for a total of 12 injections). For all five groups, the indicated therapy was carried out for six months.	PTX 400 mg orally, twice daily + penile traction therapy (PTT) + colchicine orally 0.6 mg, twice daily (for patients experiencing penile pain), for a period of 6 months.	PTX 400 mg three times + L-arginine 2 g/orally/daily. *Versus* PTX 400 mg three times + L-arginine 2 g/orally/daily + Intralesional verapamil 10 mg injections every 2 weeks (for a total of 12 injections). *Versus* Combination of the two therapies described above For all three groups, the indicated therapy was carried out for six months.	PTX 400 mg orally, twice daily + colchicine, 0.5 mg orally, twice daily + sildenafil, 50 mg orally, daily for a period of 6 months. *Versus* PTX 400 mg orally, twice daily + colchicine, 0.5 mg orally, twice daily for a period of 6 months.
Active or chronic phase	Active phase	ND	Active phase	Active phase
Follow-up period	6 months	6 months	6 months	6 months
Total number of patients	120	46	177	186
*N* treated	24	46	56	107
*N* control	24 + 24 + 24 + 24	0	76 + 45	79
Evidence for improvement? (YES/NO)				
Penile curvature	YES	YES	YES	YES
Penile pain	YES	ND	YES	YES
IIEF (Erectile function)	YES	YES	YES	YES
Penile length	ND	ND	YES	ND
Plaque size	YES	YES	ND	ND
Calcification	YES	ND	ND	ND

NOTE: PTX = pentoxifylline; ND = not defined; PTT = penile traction therapy.

**Table 4:** Characteristics of the case report studies included in the narrative review regarding treatment with PTX in patients with Peyronie’s disease.

Authors (Year) and [Reference/s]	Brant et al. [2006, Ref. [59]]	Ciociola et al. [2013, Ref. [75]]	Dell’Atti et al. [2014, Ref. [76]]	Paulis et al. [2022, Refs. [85,86,87,88]] and [2024, Ref. [89]]
Treatment	PTX 400 mg orally, three times daily/ for a period of 24 months	PTX 400 mg orally, three times daily + tadalafil 5 mg three time a week + levo-arginine 2500 mg daily + propionil-carnitine 250 mg daily + Vitamin B3 20 mg daily/ for a period of 24 months + PTT for at least 6 h daily for 1 year	PTX 400 mg orally, three times daily/ for a period of 6 months	- Combination of oral antioxidants associated with local topical therapy + perilesional penile injections with PTX 100 mg, for a period ranging from 28 to 51 months. In 7 cases (refs. [85,86,87,88]). - Combination of oral antioxidants associated with local topical therapy + perilesional penile injections with PTX 60 mg. for a period ranging from 30 to 36 months. In 3 cases (ref. [89]).
PD/Active or chronic phase	Chronic phase	Active phase	Active phase	Active phase
Follow-up period	24 months	24 months	6 months	Every 6–12 months
Duration of treatment	24 months	24 months	6 months	From 4 to 53 months
*N* treated	1	1	1	10
Evidence for improvement? (YES/NO/or not present)				
Penile curvature	YES	NO	not present	YES
Penile pain	YES	YES	not present	YES
Erectile function	YES	not present	YES	YES
Penile length	ND	YES	ND	ND
Plaque size	YES	YES	NO	YES
Calcification	YES	not present	NO	YES

NOTE: PTX = pentoxifylline; ND = not defined; PTT = penile traction therapy.

## Data Availability

All inquiries can be directed to the corresponding author.
